# Cross-cultural validation and reliability of the Leicester Cough Questionnaire in a Danish population

**DOI:** 10.1080/07853890.2026.2637260

**Published:** 2026-03-11

**Authors:** Frederik Foldager, Christine Krogsgaard Schrøder, Tatiana Jensen, Line Gangelhof Lauritsen, Janne Hastrup Jensen, Linette Marie Kofod, Jeanett Guldager Olsen, Benedicte Mechlenburg, Arietta Spinou, William Poncin, Inger Mechlenburg

**Affiliations:** ^a^ Department of Orthopaedic Surgery, Aarhus University Hospital, Aarhus, Denmark; ^b^ Department of Clinical Medicine, Aarhus University, Aarhus, Denmark; ^c^ Department of Public Health, Aarhus University, Aarhus N, Denmark; ^d^ Department of Physical Therapy and Occupational, Aarhus University Hospital, Aarhus N, Denmark; ^e^ Department of Physical and Occupational Therapy, Copenhagen University Hospital, Hvidovre, Copenhagen, Denmark; ^f^ School of Medical Sciences, Faculty of Medicine and Health, Örebro University, Sweden; ^g^ Hvidovre Municipality, Capital Region, Copenhagen, Denmark; ^h^ Department of Clinical Medicine, University of Copenhagen, Copenhagen, Denmark; ^i^ Population Health Sciences, King’s College London, London, United Kingdom; ^j^ King’s Centre for Lung Health, King’s College London, London, United Kingdom; ^k^ Pole of Pulmonology, ENT and Dermatology (LUNS), Institute of Experimental and Clinical Research (IREC), Université Catholique de Louvain (UCLouvain), Brussels, Belgium; ^l^ VIA University College, Research Centre for Health and Welfare Technology, Aarhus N, Denmark; ^m^ Department of Clinical Research, Copenhagen University Hospital, Hvidovre, Denmark

**Keywords:** Leicester Cough Questionnaire, chronic cough, reliability, internal consistency, translation, cross-cultural adaptation

## Abstract

**Background:**

Chronic cough markedly impairs health-related quality of life (HRQoL). The Leicester Cough Questionnaire (LCQ) is widely used to assess cough burden across physical, psychological, and social domains; however, no validated Danish version is available.

**Materials and methods:**

The LCQ was translated and culturally adapted into Danish in accordance with COSMIN guidelines and was approved by the original developers. Forty-two clinically stable patients with chronic cough (mean age 70.8 years; 67% male) completed baseline and retest questionnaires at a median of 28 days (IQR 21–42). Internal consistency was examined with Cronbach’s alpha. Test–retest reliability was assessed using intraclass correlation coefficients (ICC), standard error of measurement (SEM), and minimal detectable change (MDC).

**Results:**

The LCQ-DK demonstrated excellent internal consistency (Cronbach’s alpha = 0.96 total; 0.84–0.92 domains). Test–retest reliability was high (ICC = 0.90, 95% CI: 0.79–0.95 total; 0.86–0.89 domains). SEM was 1.14 points (8%) and MDC 3.16 points (23%) for the total score. No floor or ceiling effects were seen for the total score, though ceiling effects up to 12% occurred within domains.

**Conclusion:**

The LCQ-DK demonstrates high reliability following cross-cultural adaptation and supports group-level assessment of HRQoL in Danish-speaking patients with chronic cough for clinical and research use.

## Introduction

Assessing health-related quality of life (HRQoL) has become a cornerstone of modern medicine, as the field increasingly adopts a holistic approach encompassing biological, psychological, and social dimensions [1]. In this context, patient-reported outcome measures (PROMs) are recognized as essential tools in the management of chronic diseases, as they capture the direct impact of symptoms on patients’ daily lives and enable clinicians to evaluate treatment effectiveness from the patient’s perspective [2].

Chronic cough imposes significant physical, psychological, and social burdens on patients [3–5]. Its global prevalence is estimated at approximately 10% of the adults [5]. Although chronic cough is increasingly recognized as a clinically significant condition [6], its assessment remains challenging because reliable, symptom-specific instruments are needed to capture its multifaceted impact on patients.

Generic PROMs frequently fail to capture the nuanced effects of chronic cough, making disease-specific and symptom-specific tools indispensable. The Leicester Cough Questionnaire (LCQ) is a validated, multidimensional instrument designed to measure the impact of chronic cough across physical, psychological, and social domains [3]. LCQ has demonstrated very good psychometric properties, including reliability and responsiveness [3,7,8], and has been validated in patient populations where chronic cough is a prominent symptom, such as those with refractory chronic cough, bronchiectasis, chronic obstructive pulmonary disease, and cystic fibrosis [9–11].

Given its strong psychometric properties and the global prevalence of chronic cough, the availability of culturally adaptive versions of the LCQ is essential. To date, the LCQ has been translated into more than 50 languages [12], further reinforcing its widespread use as a reference instrument for assessing cough-related HRQoL across diverse settings. Relevant work reflects a broader recognition of the need for inclusive health assessments, particularly in multinational clinical research and routine care, where the use of accurate, translated, and valid PROMs is crucial [13,14].

There is currently no validated cough-specific HRQoL questionnaire in Danish. This represents a barrier to both clinical care and research, as non-validated translations may compromise the reliability of patient-reported data and hinder comparisons across patients and settings. Therefore, the cross-cultural validation of the LCQ in Danish addresses a research and clinical gap. This study will support Danish-speaking patients with chronic cough and ultimately inform local, national, and international initiatives in patient-centred respiratory care.

The objective of this study was to translate, cross-culturally validate, and assess the internal consistency and test-retest reliability of the Danish version of the Leicester Cough Questionnaire.

## Methods

### Study design and ethical approval

This study was conducted under the sponsorship of Aarhus University Hospital, as part of a research initiative launched by the Circle-U European University Alliance partners (Grant number 2023-07). In accordance with Danish national legislation, this non-interventional questionnaire-based study was exempt from review by a human research ethics committee. Approval for data protection and governance was obtained from the Danish Data Protection Agency (Approval number 1-16-02-26-24) prior to patient enrolment. The study involved forward and backward translation, as well as cross-cultural adaptation of the LCQ from English to Danish, followed by assessment of its test–retest reliability.

### The Leicester Cough Questionnaire (LCQ)

The LCQ assesses the impact of chronic cough on a patient’s HRQoL, using a 19-item questionnaire with a 7-point Likert scale. It has three domains. The physical domain includes items 1–3, 9–11, 14, and 15; the psychological domain includes items 4–6, 12, 13, 16, and 17; and the social domain includes items 7, 8, 18, and 19. Each domain score is computed as the sum of item scores divided by the number of items in that domain, resulting in a score range of 1 to 7. The total LCQ score is derived by summing the three domain scores, yielding a total score range of 3–21, with higher scores indicating better HRQoL [3].

### Translation and cross-cultural adaptation

The Consensus-based Standards for the selection of health Measurement INstruments (COSMIN) guidelines were followed for the translation and cross-cultural adaptation of the Danish version of the LCQ [15]. The original LCQ was translated from English into Danish. Two translator teams performed two independent forward translations. The first translation team consisted of two individuals: one Danish native speaker fluent in English and the other a Russian native speaker fluent in both Danish and English. Both translators had experience in healthcare, and neither had prior knowledge of respiratory disease. The second translation team consisted of two Danish native speakers who are fluent in English. One of the team’s translators had expertise in translating questionnaires and in-depth knowledge of respiratory disease, whilst the other had no experience or knowledge in these areas. After independent translations, the two translator teams collaborated to formulate the first draft of the Danish LCQ. This version was then back-translated into English by two independent translators, one native Danish speaker and the other fluent in English. One of them had prior knowledge of respiratory diseases, whilst the other one had not. Both had experience in healthcare. All six translators discussed differences between the original and back-translated versions and adjusted the Danish version of the LCQ (LCQ-DK).

The adjusted version of the LCQ-DK was pilot-tested among two native Danish speakers without respiratory disease or symptoms. The participants of the pilot test were an 87-year-old male and a 72-year-old female, who had agreed to participate in the study. They received an email containing the LCQ-DK, along with written instructions for completing it. They each returned their questionnaire responses, along with comments on the questions’ understanding and wording, *via* email. The back-translation was sent to the original developers of the questionnaire for approval, and the final version of the LCQ-DK was ready (Supplementary File 1) [3].

### Participants recruitment

All individuals were recruited from the Central Denmark Region, the Region of Southern Denmark, and the Capital Region of Denmark, having been referred for pulmonary rehabilitation. Recruitment was conducted with the assistance of physiotherapists in pulmonary departments at hospitals or in municipal settings where participants were already receiving outpatient care.

Eligible participants were adults (≥18 years) with a chronic cough, defined as a cough lasting at least 8 weeks. Participants were excluded based on the following criteria: if they did not understand or speak/write Danish, or had a cognitive impairment. Additionally, for the test-retest assessment, individuals who were in a clinically unstable phase of their disease were excluded. To minimise fluctuations in symptoms indicating clinical change between test 1 and test 2, patients completed the Borg Category Ratio scale (Borg CR 10) (Supplementary file 2) using standardised written instructions and were asked to rate the current intensity of their symptoms at rest at the time of questionnaire completion, rather than retrospectively over a longer period. Participants were excluded if the difference at rest exceeded 2 points. Patients who reported clinical improvement or deterioration were also excluded. Individuals with comorbidities were not excluded from the study to support the external validity of our results. Participation was voluntary and unpaid. All participants received oral and written information from the physiotherapists and gave verbal informed consent in accordance with the Declaration of Helsinki.

### Sample size

Sample size was determined using the hypothesis testing approach for estimating the intraclass correlation coefficient (ICC), as described by Borg et al. [16]. The aim was to test whether the true ICC exceeds a predefined minimum acceptable threshold (null hypothesis: H_0_: ρ = 0.75; alternative hypothesis: H_a_: ρ > 0.75), reflecting a high level of reliability, based on the interpretation presented in the next section [17]. Separately, for the purpose of sample size estimation, an anticipated true ICC of 0.90, which was assumed [18], with two measurements per participant (*k* = 2), a desired power of 0.80, and a significance level (α) of 0.05; the required sample size was calculated to be at least 33 participants. The normality of the differences (i.e. mean retest – mean test) was examined using quantile-quantile plots.

### Data collection

A physiotherapist recruited potential participants during their pulmonary rehabilitation program from February 2023 to February 2025. They received two sets of questionnaires, namely the LCQ-DK and the Borg CR10 [19]. They were also provided with a pre-paid return envelope and an information leaflet about the study. The information leaflet introduced the project and provided contact information for any questions. In addition, the physiotherapist provided oral information and instructions on completing the questionnaires to those who were interested. To ensure consistency, the same physiotherapist provided this information at all three recruitment sites. Patients completed and handed in the first set of questionnaires during their rehabilitation classes. They were instructed to complete the second set at least two weeks later and return it either in person to the physiotherapist during a rehabilitation session or by mail to the hospital. No information was provided regarding the maximum time for completing and returning the second set.

## Statistical analysis

### Data aggregation

The hard copies of the questionnaires were completed by hand, without support, and the data were subsequently entered into an aggregated dataset by a research assistant. Missing LCQ-DK data for more than 2 items, exacerbation of symptoms, or no LCQ retest results resulted in exclusion from the statistical analysis. The significance level was 0.05. Statistical software, Stata 18.1, ©Statacorp LLC, College Station, Texas, was used for data analysis.

### Internal consistency

Internal consistency of the LCQ-DK was assessed using Cronbach’s alpha for the analytical cohort of 42 participants who completed test and retest administrations, and met our definition of clinical stability. Cronbach’s alpha was calculated for both administrations. Cronbach’s alpha was calculated for the total score (19 items) and for each domain to evaluate the extent to which items measure the same underlying construct of HRQoL in chronic cough. Item-test and item-retest correlations were computed to identify potential issues with individual items. Cronbach’s alpha values ranging from 0.70 to 0.95 were considered acceptable, with values >0.90 indicating excellent reliability but potentially suggesting item redundancy [20].

### Test-retest reliability

Test-retest reliability was assessed using intra-class correlation coefficients (ICCs), which reflect the proportion of total variance in observed scores (σ^2^_total_) attributable to true differences between subjects (σ^2^_true_), as opposed to measurement error (σ^2^_error_), as shown in Equation 1. Based on Shrout and Fleiss’s classification, a two-way mixed-effects model for absolute agreement based on single measurements (ICC [1,2]) was applied (Equation 2) [21]. ICC values were interpreted as follows: an ICC above 0.75 indicated high reliability, 0.6 to 0.74 good reliability, 0.4 to 0.59 fair to moderate reliability, and below 0.4 poor reliability [17]. Absolute reliability was quantified using the standard error of measurement (SEM) (Equation 3) and the minimal detectable change (MDC) (Equation 4) [22]. SEM and MDC were also expressed as percentages of the grand mean (Equations 5 and 6).

(1) $$\sigma^{2}{}_{\text{total}}\;=\;\sigma^{2}{}_{\text{true}}+\sigma^{2}{}_{\text{error}}$$

(2) $${\text{ICC}}_{2.1}\;=\;\frac{\sigma_{\text{true}}^{2}}{\sigma_{\text{total}}^{2}}$$

(3) $${\text{ICC}}_{2.1}\;=\;\frac{\sigma^{2}{}_{\text{true}}}{\sigma^{2}{}_{\text{total}}}$$

(4) MDC  =  SEM  ∗  1.96  ∗  √2

(5) $${\text{SEM}}_{\%}\;=\text{ SEM}/\left(\left(\text{Mean Test }-\text{ Mean Retest}\right)/2\right)\;*\text{ 1}00$$

(6) $${\text{MDC}}_{\%}\;=\text{ MDC}/\left(\left(\text{Mean Test }-\text{ Mean Retest}\right)/2\right)\;*\text{ 1}00$$

Test–retest agreement was further examined using Bland-Altman plots, in which the mean difference (Retest – Test) with 95% confidence intervals (CIs) and 95% limits of agreement (LoA) was plotted against the average of test and retest scores ((Test + Retest)/2) [23]. The mean difference estimates potential systematic bias. If the line of equality (zero) lies within the 95% CI of the mean difference, no systematic bias is assumed. If it falls outside, this indicates a systematic bias.

### Floor and ceiling effects

The LCQ total score and its domains were evaluated for floor and ceiling effects, which are considered present if more than 15% of respondents scored at the minimum or maximum possible value, respectively [24].

## Results

### Translation and cross-cultural validation

The developers approved the back-translated LCQ-DK. Participants completing pilot tests noted that the font size was too small and requested an increase.

### Participant characteristics

**Figure 1.**
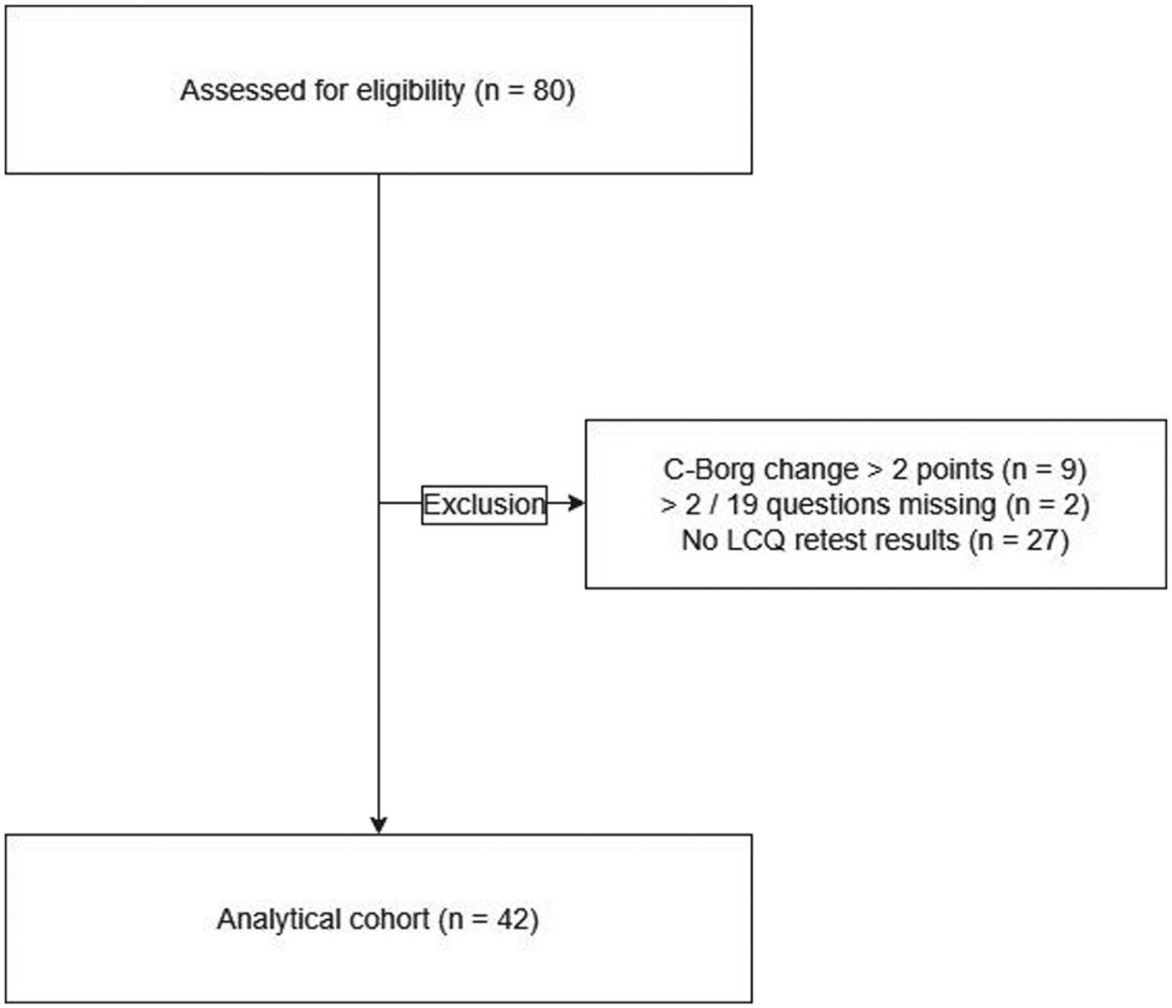
The flow diagram of patients included in the translation, cross-cultural adaptation, and test-retest results of the Leicester Cough Questionnaire (LCQ).

Of the 80 individuals assessed for eligibility, 38 were excluded due to a CR-Borg change greater than 2 points (*n* = 9), more than 2 LCQ-DK items missing (*n* = 2), or no LCQ retest results (*n* = 27), resulting in 42 participants with chronic cough being included in the test-retest analysis (Figure 1). This cohort consisted of 28 males (67%) and 14 females (33%), with a mean age of 70.8 years (SD 7.4). The mean Borg CR10 score was 2.67 (SD 2.5), and the median time between test and retest was 28 (IQR 21 to 42) days (Table 1).

**Table 1.** Characteristics of the study participants with chronic cough (*n* = 42).

Characteristic		Number of missing, n (%)
**Sex (Males/Females)**, n (%)	28 (67%) / 14 (33%)	0 (0%)
**Age in years**, mean (SD)	70.8 (7.4)	22 (52%)
**Borg-CR 10 (0–10)**, mean (SD)	2.67 (2.5)	0 (0%)
**Hospital/municipality, n (%)**		
Aarhus	23 (55%)	0 (0%)
Hvidovre	18 (43%)	
Kolding	1 (2%)	
**Time between test and retest (days)**, mean (SD)	33.5 (17.6)	12 (28.6%)

N: Number of participants. SD: Standard Deviation. Borg-CR10: Borg Category-Ratio 10 Scale.

### Internal consistency

The LCQ-DK demonstrated excellent internal consistency, with a Cronbach’s alpha of 0.96 (95% CI 0.94; 0.98) for the total score at both the test and retest administrations (Table 2). For the physical domain, Cronbach’s alpha was 0.90 at both test and retest. For the psychological domain, Cronbach’s alpha was 0.92 at test and 0.91 at retest, and for the social domain, Cronbach’s alpha was 0.84 at test and 0.89 at retest, indicating that items within each domain reliably measure the impact of chronic cough on health-related quality of life. Item–test correlations ranged from 0.57 to 0.93 for the test administration and from 0.46 to 0.92 for the retest administration, with lower correlations observed for item 9 (0.58 test; 0.69 retest), item 15 (0.57 test; 0.46 retest), and item 19 (0.57 test; 0.79 retest). There were no missing items; therefore, no item-level analysis of missing data was required.

**Table 2.** Internal consistency of the Leicester Cough Questionnaire (LCQ) (*n* = 42), with item and domain-level Cronbach’s alpha for test and retest administration.

	Test administration			Retest administration		
Item	Item-Test Correlation	Item-Rest Correlation	Alpha, if item-removed	Item-Test Correlation	Item-Rest Correlation	Alpha, if item-removed
Question 1	0.76	0.73	0.96	0.83	0.82	0.96
Question 2	0.72	0.68	0.96	0.69	0.66	0.96
Question 3	0.88	0.86	0.96	0.92	0.91	0.96
Question 4	0.63	0.58	0.96	0.71	0.67	0.96
Question 5	0.87	0.85	0.96	0.79	0.76	0.96
Question 6	0.82	0.80	0.96	0.73	0.70	0.96
Question 7	0.81	0.79	0.96	0.88	0.87	0.96
Question 8	0.93	0.91	0.96	0.86	0.84	0.96
Question 9	0.58	0.53	0.96	0.69	0.66	0.96
Question 10	0.78	0.75	0.96	0.86	0.85	0.96
Question 11	0.87	0.85	0.96	0.76	0.73	0.96
Question 12	0.86	0.85	0.96	0.90	0.88	0.96
Question 13	0.88	0.86	0.96	0.90	0.89	0.96
Question 14	0.75	0.72	0.96	0.79	0.76	0.96
Question 15	0.57	0.52	0.96	0.46	0.40	0.97
Question 16	0.73	0.70	0.96	0.74	0.70	0.96
Question 17	0.85	0.82	0.96	0.75	0.72	0.96
Question 18	0.80	0.77	0.96	0.80	0.78	0.96
Question 19	0.57	0.51	0.96	0.79	0.76	0.96
**Domain**						
Physical (95% CI)			0.90 (0.84; 0.96)			0.90 (0.84; 0.97)
Psychological (95% CI)			0.92 (0.88; 0.96)			0.91 (0.86; 0.96)
Social burden (95% CI)			0.84 (0.75; 0.93)			0.89 (0.80; 0.98)
**Total score**						
Total			0.96 (0.94; 0.98)			0.96 (0.94; 0.98)

LCQ: Leicester Cough Questionnaire. 95% CI: 95% Confidence Interval.

### Test-retest reliability

**Figure 2.**
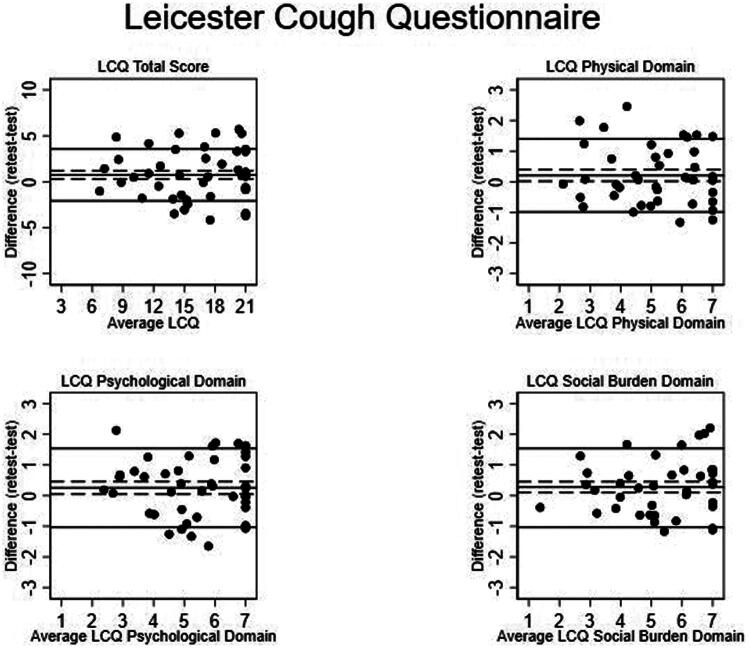
Bland-Altman plots for the agreement between test and retest scores for the Leicester Cough Questionnaire (LCQ) total scores and each domain. Bland and Altman plots present the agreement between test and retest for the LCQ questionnaire total score and for each domain. The Y-axis shows the difference between the two paired measurements (retest-test), and the X-axis shows the average of these measurements (retest + test)/2). The mean difference is marked with a horizontal thick black line, and the surrounding dashed lines represent the 95% confidence interval of the mean difference. The upper and lower 95% limits of agreement (mean difference ± 1.96 * SD) are marked with thick lines.

The difference between the test and retest for the LCQ total and domain scores is reported in Table 3 and Figure 2. The mean difference in the LCQ total score was 0.74 points (95% CI 0.29; 1.19), and the LoA was −2.09 to 3.57. The mean difference in the physical domain was 0.21 points (95% CI 0.16; 0.40), and the LoA was −0.99 to 1.40. The mean difference in the psychological domain was 0.28 points (95% CI 0.10; 0.46), and the LoA was −1.03 to 1.53. The mean difference in the social domain was 0.26 points (95% CI 0.05; 0.46), and the LoA was −1.03 to 1.53.

**Table 3.** Measures of test-retest reliability of the Leicester Couch Questionnaire (LCQ) in participants with chronic cough (*n* = 42).

Variable	Test Mean (SD)	Retest Mean (SD)	Difference Mean (95% CI)^a^	ICC [2.1] (95% CI)	SEM (%)	MDC (%)
LCQ-DK Total score	15.8 (3.7)	16.5 (3.6)	0.74 (0.29; 1.19)	0.90 (0.79; 0.95)	1.14 (8)	3.16 (23)
**Domains**						
1. Physical	5.09 (1.21)	5.29 (1.15)	0.21 (0.16; 0.40)	0.86 (0.74; 0.92)	0.45 (10)	1.25 (28)
2. Psychological	5.35 (1.33)	5.60 (1.29)	0.26 (0.05; 0.46)	0.86 (0.74; 0.93)	0.49 (11)	1.37 (30)
3. Social burden	5.35 (1.30)	5.63 (1.29)	0.28 (0.10; 0.46)	0.89 (0.76; 0.94)	0.44 (10)	1.23 (27)

LCQ: Leicester Cough Questionnaire. ICC: Intra-Class Correlation. SEM: Standard Error of Measurement. SEM%: Standard Error of Measurement in percent of the grand mean. MDC: Minimal Detectable Change. MDC%: Minimal Detectable Change in percent of the grand mean. 95% CI: 95% Confidence Interval.

^a^ Calculated as retest–test.

The ICC coefficient for the LCQ total score was 0.90 (95% CI: 0.79; 0.95), with an SEM of 1.14 points, corresponding to a SEM_%_ of 8%, and a MDC of 3.16 points, corresponding to a MDC_%_ of 23%. The ICC coefficients for the domains of the LCQ ranged from 0.86 (95% CI: 0.74; 0.92) for the physical and psychological domains to 0.89 (95% CI: 0.76; 0.94) for the social burden domain. The SEM for the domains ranged from 0.44 to 0.49, corresponding to SEM_%_ of 10% to 11%. The MDC ranged from 1.23 points to 1.37 points, corresponding to an MDC_%_ of 27% to 30% (Table 3).

### Floor and ceiling effects

The LCQ total score exhibited no floor or ceiling effects (0% for both), while the individual domains showed no floor effects across all domains (0%) and ceiling effects ranging from 0% for the physical and social burden domains to 12% for the psychological domain.

## Discussion

This study evaluated the translation, cross-cultural adaptation, internal consistency, and test–retest reliability of the LCQ-DK. The LCQ-DK demonstrated performance comparable to the original instrument and previously published translations. In addition to high relative reliability, the study provides absolute reliability estimates, including SEM and MDC, supporting interpretation of change beyond random measurement error at the group level in Danish-speaking patients with chronic cough.

Internal consistency was high for the total score and across domains, consistent with the original validation and most cross-cultural adaptations. Previous studies have generally reported Cronbach’s alpha values exceeding accepted thresholds, with several translations reporting total scores above 0.90 [25]. While very high alpha values may indicate overlapping item content, item-level analyses in the present study suggested that only a small number of items contributed less strongly to overall coherence. This pattern supports the robustness of the LCQ-DK while indicating potential scope for future evaluation of item efficiency rather than deficiencies in scale performance.

Test–retest reliability was high and consistent with findings reported across other LCQ translations. A recent systematic review identified ICCs ranging from 0.71 to 0.96 for the total score and from 0.70 to 0.93 for individual domains in studies of acceptable methodological quality, with overall evidence rated as moderate according to COSMIN criteria [25]. In this context, the LCQ-DK demonstrated reliability well within the range observed across languages and clinical populations.

Although group-level reliability was supported by high ICC values, Bland–Altman analyses indicated greater variability in individual test–retest differences, with increasing spread at higher scores. This finding suggests limited interchangeability of individual measurements and highlights the need for caution when interpreting individual-level change, particularly when retest intervals are long or variable. Agreement metrics were broadly comparable to those previously reported, with both mean differences and limits of agreement varying substantially across studies [25].

The present study also provides absolute reliability estimates, including SEM and MDC, which have not been reported in prior LCQ studies. These metrics provide additional insight into measurement precision and thresholds for distinguishing change beyond random measurement error.

No floor or ceiling effects were observed in the total score, indicating an appropriate distribution across the measured range. A modest ceiling effect was observed in the psychological domain, although the proportion of participants scoring at the maximum remained below commonly used thresholds [24]. Overall, these findings suggest that the LCQ-DK captures a broad spectrum of cough-related quality-of-life impact.

### Strengths and limitations

Strengths of this study include a structured cross-cultural adaptation process, evaluation of both relative and absolute reliability, and complementary assessment of agreement. Several limitations should be considered. Pilot testing during the translation phase was conducted in two native Danish speakers without respiratory disease and focused primarily on linguistic clarity. While appropriate for the intended purpose, inclusion of patients with chronic cough and individuals with more diverse educational and cultural backgrounds could have further strengthened the assessment of comprehensibility and relevance.

Although the sample size exceeded the minimum required for hypothesis testing of reliability, it was smaller than the COSMIN recommendation to achieve narrow confidence intervals around ICC estimates, thereby limiting precision [26]. All analysed participants had a chronic cough and were required to be clinically stable for test–retest assessment. While this was necessary to evaluate reliability, the limited heterogeneity of the sample restricts inference to other respiratory populations and precludes evaluation across the full spectrum of cough-related conditions.

The test–retest interval was relatively long and variable, and incomplete interval data limited full characterisation of timing for all participants. This may have allowed genuine symptom change in some individuals despite efforts to ensure clinical stability.

Missing data warrants consideration. Several eligible participants were excluded because of clinically relevant changes on the Borg CR10 scale or non-completion of the retest questionnaire. While these exclusions ensured clinical stability in the analysed sample, the resulting attrition may introduce selection or non-response bias and should be considered when interpreting generalisability.

The study did not assess several psychometric properties beyond its primary scope. Construct validity, responsiveness, and dimensional structure were not evaluated because no comparator instruments were included, participants were required to be clinically stable, and the sample size was insufficient for factor analysis.

### Future research

Future Danish studies should evaluate responsiveness and minimal important change using patient-anchored methods, assess dimensional structure in larger, more heterogeneous samples, and examine agreement patterns across shorter, more standardised retest intervals. Additional work may also explore whether selected items contribute limited information in Danish settings and whether refined versions of the LCQ could improve feasibility without compromising measurement performance.

## Conclusion

This study demonstrates that the Danish version of the Leicester Cough Questionnaire, following a rigorous cross-cultural adaptation process, shows high internal consistency and strong test–retest reliability, with acceptable measurement error and no floor or ceiling effects. The findings support the use of the LCQ-DK for group-level assessment of cough-related HRQoL in Danish-speaking patients with chronic cough, while individual-level change should be interpreted with caution. Further studies are needed to evaluate responsiveness and minimal important changes in clinical populations experiencing treatment effects or disease progression.

## Supplementary Material

Original Leicester Cough Questionnaire.pdf

Supplementary files.docx

## Data Availability

Due to the Data Use Agreement with the Danish Data Protection Agency (Journal No. 1-16-02-26-24), individual-level data cannot be shared. Aggregated, anonymised data underlying the results and related analysis code are available from the corresponding author upon reasonable request.

## References

[1] Kharroubi SA, Elbarazi I. Editorial: health-related quality of life in health care. Front Public Health. 2023;11:1123180. doi: 10.3389/fpubh.2023.1123180. 36817896 PMC9932954

[2] Churruca K, Pomare C, Ellis LA, et al. Patient-reported outcome measures (PROMs): a review of generic and condition-specific measures and a discussion of trends and issues. Health Expect. 2021;24(4):1015–1024. doi: 10.1111/hex.13254. 33949755 PMC8369118

[3] Birring SS, Prudon B, Carr AJ, et al. Development of a symptom specific health status measure for patients with chronic cough: Leicester Cough Questionnaire (LCQ). Thorax. 2003;58(4):339–343. doi: 10.1136/thorax.58.4.339. 12668799 PMC1746649

[4] Brignall K, Jayaraman B, Birring SS. Quality of life and psychosocial aspects of cough. Lung. 2008;186 (Suppl 1):S55–S8. doi: 10.1007/s00408-007-9034-x. 17939003

[5] Song W-J, Chang Y-S, Faruqi S, et al. The global epidemiology of chronic cough in adults: a systematic review and meta-analysis. Eur Respir J. 2015;45(5):1479–1481. doi: 10.1183/09031936.00218714. 25657027

[6] Morice AH, Millqvist E, Bieksiene K, et al. ERS guidelines on the diagnosis and treatment of chronic cough in adults and children. Eur Respir J. 2020;55(1):1901136. doi: 10.1183/13993003.01136-2019. 31515408 PMC6942543

[7] Birring SS, Matos S, Patel RB, et al. Cough frequency, cough sensitivity and health status in patients with chronic cough. Respir Med. 2006;100(6):1105–1109. doi: 10.1016/j.rmed.2005.09.023. 16266801

[8] Raj AA, Pavord DI, Birring SS. Clinical cough IV:what is the minimal important difference for the Leicester Cough Questionnaire? Handb Exp Pharmacol. 2009;(187):311–320. doi: 10.1007/978-3-540-79842-2_16. 18825348

[9] Berkhof FF, Boom LN, ten Hertog NE, et al. The validity and precision of the Leicester Cough Questionnaire in COPD patients with chronic cough. Health Qual Life Outcomes. 2012;10(1):4. doi: 10.1186/1477-7525-10-4. 22230731 PMC3311606

[10] Murray MP, Turnbull K, MacQuarrie S, et al. Validation of the Leicester Cough Questionnaire in non-cystic fibrosis bronchiectasis. Eur Respir J. 2009;34(1):125–131. doi: 10.1183/09031936.00160508. 19196812

[11] Ward N, Stiller K, Rowe H, et al. The psychometric properties of the Leicester Cough Questionnaire and Respiratory Symptoms in CF tool in cystic fibrosis: A preliminary study. J Cyst Fibros. 2017;16(3):425–432. doi: 10.1016/j.jcf.2016.11.011. 27986494

[12] Turner RD, Birring SS. Measuring cough: what really matters? J Thorac Dis. 2023;15(4):2288–2299. doi: 10.21037/jtd-23-230. 37197542 PMC10183488

[13] Grant SR, Noticewala SS, Mainwaring W, et al. Non-English language validation of patient-reported outcome measures in cancer clinical trials. Support Care Cancer. 2020;28(6):2503–2505. doi: 10.1007/s00520-020-05399-9. 32189098

[14] Slade AL, Retzer A, Ahmed K, et al. Systematic review of the use of translated patient-reported outcome measures in cancer trials. Trials. 2021;22(1):306. doi: 10.1186/s13063-021-05255-z. 33902699 PMC8074490

[15] Mokkink LB, Prinsen CAC, Patrick DL, et al. COSMIN Study Design checklist for Patient-reported outcome measurement instruments [Internet]. Amsterdam: COSMIN initiative; 2019 [cited 2026 Feb 26]. Available from: https://www.cosmin.nl.

[16] Borg DN, Bach AJE, O’Brien JL, et al. Calculating sample size for reliability studies. Pm R. 2022;14(8):1018–1025. doi: 10.1002/pmrj.12850. 35596122

[17] McDowell I. Measuring health: a guide to rating scales and questionnaires. 3rd ed. New York: Oxford University Press; 2006.

[18] Ternesten-Hasséus E, Johansson E-L. Validity and reliability of the Swedish version of the Leicester Cough Questionnaire in unexplained chronic cough. Respir Med. 2024;224:107582. doi: 10.1016/j.rmed.2024.107582. 38428509

[19] Crisafulli E, Clini EM. Measures of dyspnea in pulmonary rehabilitation. Multidiscip Respir Med. 2010;5(3):202–210. doi: 10.1186/2049-6958-5-3-202. 22958431 PMC3463047

[20] Tavakol M, Dennick R. Making sense of Cronbach’s alpha. Int J Med Educ. 2011;2:53–55. doi: 10.5116/ijme.4dfb.8dfd. 28029643 PMC4205511

[21] Weir JP. Quantifying test-retest reliability using the intraclass correlation coefficient and the sem. J Strength Cond Res. 2005;19(1):231–240. doi: 10.1519/15184.1. 15705040

[22] Bland JM, Altman DG. Measurement error. BMJ. 1996;312(7047):1654. doi: 10.1136/bmj.313.7059.744. 8664723 PMC2351401

[23] Bland JM, Altman DG. Statistical methods for assessing agreement between two methods of clinical measurement. Int J Nurs Stud. 2010;47(8):931–936. doi: 10.1016/j.ijnurstu.2009.10.001. 2868172

[24] Terwee CB, Bot SDM, de Boer MR, et al. Quality criteria were proposed for measurement properties of health status questionnaires. J Clin Epidemiol. 2007;60(1):34–42. doi: 10.1016/j.jclinepi.2006.03.012. 17161752

[25] Bottine A, Grandjean J, Standaert M, et al. A systematic review of the psychometric properties of the Leicester Cough Questionnaires based on the COSMIN guidelines. Respir Med. 2024;231:107739. doi: 10.1016/j.rmed.2024.107739. 39029808

[26] Terwee CB, Mokkink LB, Knol DL, et al. Rating the methodological quality in systematic reviews of studies on measurement properties: a scoring system for the cosmin checklist. Qual Life Res. 2012;21(4):651–657. doi: 10.1007/s11136-011-9960-1. 21732199 PMC3323819

