# Supplementary material for: Cross-cultural validation and reliability of the Leicester Cough Questionnaire in a Danish population
Source: Ann Med. 2026 Mar 11;58(1):2637260. doi: 10.1080/07853890.2026.2637260 (PMC12983802; doi:10.1080/07853890.2026.2637260)
Supplement: Original Leicester Cough Questionnaire.pdf [file IANN_A_2637260_SM4376.pdf]

stradOS®

Leicester Cough Questionnaire (LCQ)

INSTRUCTIONS: These questions are designed to assess the impact of your cough on various aspects of your life. Read each question carefully and answer by checking the response that best applies to you in the past **TWO WEEKS**

		All the time	Most of the time	A good bit of the time	Some of the time	A little bit of the time	Hardly any of the time	None of the time
1	Have you had chest or stomach pains as a result of your cough?	1	2	3	4	5	6	7
2	Have you been bothered by phlegm production when you cough?	1	2	3	4	5	6	7
3	Have you been tired because of your cough?	1	2	3	4	5	6	7
4	Have you felt in control of your cough?	7	6	5	4	3	2	1
5	Have you felt embarrassed by your coughing?	1	2	3	4	5	6	7
6	My cough has made me feel anxious	1	2	3	4	5	6	7
7	My cough has interfered with my job, or other daily tasks	1	2	3	4	5	6	7
8	I felt that my cough interfered with the overall enjoyment of my life	1	2	3	4	5	6	7
9	Exposure to paint or fumes has made me cough	1	2	3	4	5	6	7
10	Has your cough disturbed your sleep?	1	2	3	4	5	6	7
11	How many times a day have you had coughing bouts?	1	2	3	4	5	6	7
12	My cough has made me feel frustrated	1	2	3	4	5	6	7

stradOS®

**13**	My cough has made me feel fed up	1	2	3	4	5	6	7
**14**	Have you suffered from a hoarse voice as a result of your cough?	1	2	3	4	5	6	7
**15**	Have you had a lot of energy?	7	6	5	4	3	2	1
**16**	Have you worried that your cough may indicate a serious illness?	1	2	3	4	5	6	7
**17**	Have you been concerned that other people think something is wrong with you, because of your cough?	1	2	3	4	5	6	7
**18**	My cough has interrupted conversations or telephone calls	1	2	3	4	5	6	7
**19**	I feel that my cough has annoyed my partner, family or friends.	1	2	3	4	5	6	7
