# Supplementary material for: Cross-cultural validation and reliability of the Leicester Cough Questionnaire in a Danish population
Source: Ann Med. 2026 Mar 11;58(1):2637260. doi: 10.1080/07853890.2026.2637260 (PMC12983802; doi:10.1080/07853890.2026.2637260)
Supplement: Supplementary files.docx [file IANN_A_2637260_SM4373.docx]

**Supplementary file 1.** Leicester Cough Questionnaire, Danish version (LCQ-DK).

**Spørgeskema om hoste fra Leicester**

Dette spørgeskema er designet til at vurdere din hostes påvirkning på forskellige aspekter af dit liv, Læs hvert spørgsmål omhyggeligt og sæt en cirkel omkring det svar, der passer bedst på dig. Svar venligst på ALLE spørgsmål så godt, som du kan.

**1. Har du inden for de seneste 2 uger haft bryst- eller mavesmerter som følge af din hoste?**

| 1 | 2 | 3 | 4 | 5 | 6 |  |
| --- | --- | --- | --- | --- | --- | --- |
| Hele tiden | Det meste af tiden | En stor del af tiden | Noget af tiden | Nogle gange | Sjældent | Aldrig |

**2. Har du inden for de seneste 2 uger været generet af slim, når du hoster?**

| 1 | 2 | 3 | 4 | 5 | 6 | 7 |
| --- | --- | --- | --- | --- | --- | --- |
| Hele tiden | Det meste af tiden | En stor del af tiden | Noget af tiden | Nogle gange | Sjældent | Aldrig |

**3. Har du inden for de seneste 2 uger været træt på grund af din hoste?**

| 1 | 2 | 3 | 4 | 5 | 6 | 7 |
| --- | --- | --- | --- | --- | --- | --- |
| Hele tiden | Det meste af tiden | En stor del af tiden | Noget af tiden | Nogle gange | Sjældent | Aldrig |

**4. Har du inden for de seneste 2 uger følt, at du havde kontrol over din hoste?**

| 1 | 2 | 3 | 4 | 5 | 6 | 7 |
| --- | --- | --- | --- | --- | --- | --- |
| Aldrig | Sjældent | Nogle gange | Noget af tiden | En stor del af tiden | Det meste af tiden | Hele tiden |

**5. Hvor ofte i løbet af de seneste 2 uger har du følt dig pinligt berørt over din hoste?**

| 1 | 2 | 3 | 4 | 5 | 6 | 7 |
| --- | --- | --- | --- | --- | --- | --- |
| Hele tiden | Det meste af tiden | En stor del af tiden | Noget af tiden | Nogle gange | Sjældent | Aldrig |

**6. I de seneste 2 uger har min hoste gjort mig ængstelig**.

| 1 | 2 | 3 | 4 | 5 | 6 | 7 |
| --- | --- | --- | --- | --- | --- | --- |
| Hele tiden | Det meste af tiden | En stor del af tiden | Noget af tiden | Nogle gange | Sjældent | Aldrig |

**7. I de seneste 2 uger har min hoste forstyrret mit arbejde eller andre daglige gøremål.**

| 1 | 2 | 3 | 4 | 5 | 6 | 7 |
| --- | --- | --- | --- | --- | --- | --- |
| Hele tiden | Det meste af tiden | En stor del af tiden | Noget af tiden | Nogle gange | Sjældent | Aldrig |

**8. I de seneste 2 uger har jeg følt, at min hoste forstyrrede min generelle livsglæde.**

| 1 | 2 | 3 | 4 | 5 | 6 | 7 |
| --- | --- | --- | --- | --- | --- | --- |
| Hele tiden | Det meste af tiden | En stor del af tiden | Noget af tiden | Nogle gange | Sjældent | Aldrig |

**9. I de seneste 2 uger har udsættelse for maling eller dampe fået mig til at hoste.**

| 1 | 2 | 3 | 4 | 5 | 6 | 7 |
| --- | --- | --- | --- | --- | --- | --- |
| Hele tiden | Det meste af tiden | En stor del af tiden | Noget af tiden | Nogle gange | Sjældent | Aldrig |

**10. Har din hoste forstyrret din søvn indenfor de seneste 2 uger?**

| 1 | 2 | 3 | 4 | 5 | 6 | 7 |
| --- | --- | --- | --- | --- | --- | --- |
| Hele tiden | Det meste af tiden | En stor del af tiden | Noget af tiden | Nogle gange | Sjældent | Aldrig |

**11. Hvor mange gange om dagen har du haft hosteanfald I de seneste 2 uger?**

| 1 | 2 | 3 | 4 | 5 | 6 | 7 |
| --- | --- | --- | --- | --- | --- | --- |
| Hele tiden kontinuerligt | Det meste af tiden i løbet af dage | Flere gange i løbet af dagen | Nogle gange i løbet af dagen | Få gange i løbet af dagen | Sjældent | Aldrig |

**12. I de seneste 2 uger har min hoste gjort mig frustreret.**

| 1 | 2 | 3 | 4 | 5 | 6 | 7 |
| --- | --- | --- | --- | --- | --- | --- |
| Hele tiden | Det meste af tiden | En stor del af tiden | Noget af tiden | Nogle gange | Sjældent | Aldrig |

**13. I de seneste 2 uger har jeg følt mig træt af det på grund af min hoste.**

| 1 | 2 | 3 | 4 | 5 | 6 | 7 |
| --- | --- | --- | --- | --- | --- | --- |
| Hele tiden | Det meste af tiden | En stor del af tiden | Noget af tiden | Nogle gange | Sjældent | Aldrig |

**14. Har du inden for de seneste 2 uger oplevet at have en hæs stemme på grund af din hoste?**

| 1 | 2 | 3 | 4 | 5 | 6 | 7 |
| --- | --- | --- | --- | --- | --- | --- |
| Hele tiden | Det meste af tiden | En stor del af tiden | Noget af tiden | Nogle gange | Sjældent | Aldrig |

**15. Har du inden for de seneste 2 uger haft meget energi?**

| 1 | 2 | 3 | 4 | 5 | 6 | 7 |
| --- | --- | --- | --- | --- | --- | --- |
| Aldrig | Sjældent | Nogle gange | Noget af tiden | En stor del af tiden | Det meste af tiden | Hele tiden |

**16. Har du inden for de seneste 2 uger været bekymret for, at din hoste kunne være tegn på alvorlig sygdom?**

| 1 | 2 | 3 | 4 | 5 | 6 | 7 |
| --- | --- | --- | --- | --- | --- | --- |
| Hele tiden | Det meste af tiden | En stor del af tiden | Noget af tiden | Nogle gange | Sjældent | Aldrig |

**17. Har du inden for de seneste 2 uger været bekymret for, at andre mennesker tror, at der er noget galt med dig på grund af din hoste?**

| 1 | 2 | 3 | 4 | 5 | 6 | 7 |
| --- | --- | --- | --- | --- | --- | --- |
| Hele tiden | Det meste af tiden | En stor del af tiden | Noget af tiden | Nogle gange | Sjældent | Aldrig |

**18. Har du inden for de seneste 2 uger måtte afbryde en samtaler eller telefonsamtale på grund af hoste?**

| 1 | 2 | 3 | 4 | 5 | 6 | 7 |
| --- | --- | --- | --- | --- | --- | --- |
| Hver gang | De fleste gange | En stor del af gangene | Nogle gange | Få gange | Stort set aldrig | Aldrig |

**19. I de seneste 2 uger har jeg følt, at min hoste har irriteret min partner, familie eller venner.**

| 1 | 2 | 3 | 4 | 5 | 6 | 7 |
| --- | --- | --- | --- | --- | --- | --- |
| Hver gang, når jeg hoster | De fleste gange, når jeg hoster | En stor del af gangene, når jeg hoster | Nogle gange, når jeg hoster | Få gange, når jeg hoster | Stort set aldrig | Aldrig |

**Tak, fordi du har udfyldt dette spørgeskema!**

**Supplementary File 2.** Borg CR 10 Scale.
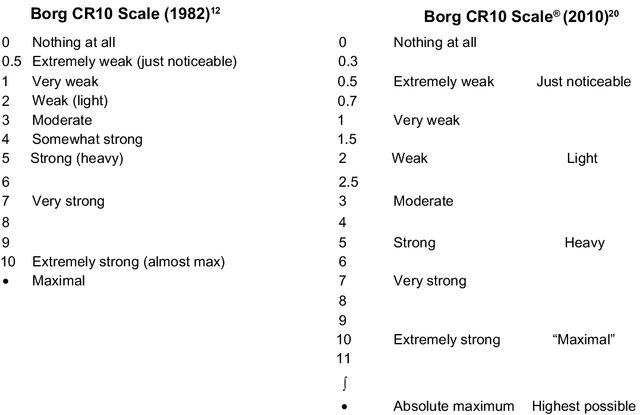


The original Borg CR10 Scale ® used to measure the perception of intensity of any experience compared to the Borg CR10 ® Scale in the recent Borg CR Scales folder. The Borg CR10 Scale ® with instructions can be obtained for a minor fee from Borg Perception, Rädisvägen 124, S-16573, Hässelby, Sweden.
